# Radioembolization Is a Safe and Effective Treatment for Hepatocellular Carcinoma with Portal Vein Thrombosis: A Propensity Score Analysis

**DOI:** 10.1371/journal.pone.0154986

**Published:** 2016-05-05

**Authors:** Young Youn Cho, Minjong Lee, Hyo-Cheol Kim, Jin Wook Chung, Yun Hwan Kim, Geum-Youn Gwak, Si Hyun Bae, Do Young Kim, Jeong Heo, Yoon Jun Kim

**Affiliations:** 1 Department of Internal Medicine and Liver Research Institute, Seoul National University College of Medicine, Seoul, Korea; 2 Department of Radiology, Seoul National University College of Medicine, Seoul, Korea; 3 Department of Radiology, College of Medicine, Korea University, Anam Hospital, Seoul, Korea; 4 Department of Medicine, Samsung Medical Center, Sungkyunkwan University School of Medicine, Seoul, Korea; 5 Department of Internal Medicine, Seoul St. Mary's Hospital, The Catholic University of Korea, Seoul, Korea; 6 Department of Internal Medicine, Yonsei University College of Medicine, Seoul, Korea; 7 Internal Medicine, Pusan National University College of Medicine, Pusan, Korea; University of Navarra School of Medicine and Center for Applied Medical Research (CIMA), SPAIN

## Abstract

**Background/Aims:**

Limited treatment options are available for patients with hepatocellular carcinoma (HCC) with portal vein thrombosis (PVT). Transarterial radioembolization using Yttrium-90 microspheres is a new treatment modality for HCC with PVT. For this analysis, we compared responses to treatment with radioembolization and with sorafenib.

**Methods:**

We evaluated 32 patients who were part of a multicenter retrospective cohort. All patients had PVT without extrahepatic metastasis and were treated with radioembolization in one of six tertiary referral hospitals in Korea. We retrospectively enrolled another 31 consecutive PVT patients without extrahepatic metastasis from a single center who received sorafenib treatment to serve as the control group. We used inverse probability weighting (IPW) using propensity scores to adjust for the between-group differences in baseline characteristics.

**Results:**

At 3 months, the response rate and disease control rate were 32.1% (9/32) and 57.1% (16/32), respectively, in the radioembolization group and 3.2% (1/31) and 41.9% (13/31) in the sorafenib group. Median overall survival (OS) and time to progression (TTP) were not significantly different between the radioembolization group and the sorafenib group (13.8 months and 10.0 months, P = 0.22; and 6.0 months and 6.0 months, P = 0.08; respectively). No differences in OS (P = 0.97) or TTP (P = 0.34) were observed after IPW was applied to balance the population characteristics. The sorafenib group showed significantly more grade 3/4 adverse effects than the radioembolization group (P < 0.01).

**Conclusion:**

HCC patients with PVT who underwent radioembolization achieved comparable survival to patients who received sorafenib, even after application of IPW. The radioembolization group also experienced fewer severe adverse effects. Radioembolization can be considered a new treatment option for patients with HCC with PVT.

## Introduction

In 2008, hepatocellular carcinoma (HCC) was the sixth most common cancer and the third most frequent cause of death worldwide [1]. Patients with vascular invasion account for a large proportion of HCC cases; these patients are classified as having advanced stage HCC, according to the Barcelona Clinic Liver Cancer (BCLC) staging system. Portal vein thrombosis (PVT) is the main cause of vascular invasion in HCC patients and negatively affects prognosis [2]. Definitive treatment such as liver transplantation (LT) is contraindicated in HCC patients with PVT because patients with PVT have high recurrence rates and low cure rates. Also, PVT at the time of LT is associated with high mortality and graft failure [3]. The BCLC classification and western guidelines [4, 5] recommend sorafenib as the treatment of choice for HCC with PVT on the basis of large phase III trials. However, sorafenib has low disease control rates and offers only minor gains in overall survival (OS) [6, 7]. Neither the Sorafenib Hepatocellular Carcinoma Assessment Randomized Protocol (SHARP) trial [8] nor the Asia-Pacific trial [9] reported any cases of complete remission (CR) and reported only minor partial remission (PR) after treatment with sorafenib. Those trials provide evidence to support sorafenib use for HCC patients with PVT, but the low response rates are unsatisfactory for most clinicians.

HCC patients with PVT require novel treatments, and, since the cause of death for most patients with advanced HCC is intrahepatic progression rather than complication from metastatic disease, locoregional treatments such as transarterial chemoembolization (TACE) or hepatic resection have been widely performed in Asian countries [10]. Several retrospective studies of hepatic resection performed in HCC patients with PVT showed long term survival [11–13]. Surgical resection is the only treatment that can achieve cure in this population, and it has potential benefits such as decreased portal pressure and improved quality of life. Still, PVT of the main portal vein showed a dismal prognosis in the previous studies, and patients must be carefully selected for surgical resection. TACE is not recommended by western guidelines, but clinicians widely use TACE for treatment of PVT; TACE is included in the consensus recommendations of the Asia-Pacific Association for the Study of the Liver [14]. A prospective non-randomized study showed that TACE increased median survival compared to conservative treatment [15], and recent retrospective studies showed that TACE achieved comparable outcomes to sorafenib [16]. However, TACE poses a risk of embolic side effects such as ischemic hepatic injuries, and treatment with TACE is contraindicated for most patients with main PVT.

Transarterial radioembolization using Yttrium-90 (Y90) is one of the most promising tools for treating HCC with PVT. Radioembolization delivers Y90-loaded microspheres into tumor-feeding arteries using similar vascular access techniques as those used in TACE. The injected microspheres emit limited radiation (mean range from the microsphere, 2.5 mm) and kill the tumor cells. Compared to sorafenib, radioembolization has a higher chance of CR, and, in some cases, successful down-staging with radioembolization can lead to curative treatments [17]. Further, radioembolization has a more tolerable side effect profile than sorafenib [18, 19]. Radioembolization also demonstrates longer time to progression (TTP) and lower rates of toxicity than TACE [20]. Radioembolization has similar OS in intermediate stage HCC compared to sorafenib [21]. The theoretical advantage of performing radioembolization instead of TACE in HCC with PVT is that radioembolization has a lower risk of ischemic side effects due to minimal arterial occlusion [22].

Few studies have investigated the survival profiles of radioembolization in HCC with PVT [23–25], a recent study compared radioembolization and sorafenib for intermediate to locally advanced HCC and concluded that radioembolization and sorafenib might have comparable outcomes [19], and another recent study compared radioembolization and sorafenib with PVT and concluded that radioembolization has favorable outcome compared to sorafenib [26]. However, more evidences are needed for comparison of radioembolization and sorafenib in the treatment of HCC with PVT. The aim of this study was to evaluate treatment outcomes of radioembolization in patients with HCC with PVT and to compare the outcomes with sorafenib treatment.

## Materials and Methods

### Patient selection

We selected patients with BCLC stage C HCC with PVT from a multicenter retrospective cohort to investigate the effect of radioembolization in patients with intermediate or advanced HCC. Patients underwent radioembolization from January 2008 to December 2013. We retrospectively enrolled consecutive HCC patients with PVT treated with sorafenib during the same period at a single center. The study protocol conformed to the ethical guidelines of the World Medical Association Declaration of Helsinki and was approved by the Institutional Review Board (IRB) of Seoul National University Hospital, and also approved by the IRB of all the other centers (Korea University, Sungkyunkwan University, The Catholic University of Korea, Yonsei University, Pusan National University), and informed consent was waived. Patient information was anonymized and de-identified prior to analysis. There was a previous Korean prospective study evaluating the efficacy of radioembolization [27], the retrospective cohort of our study was not based on the previous prospective study, but due to the retrospective nature of the cohort there may be some overlap in the study population.

The inclusion criteria were: (a) HCC patients with PVT, (b) age of at least 18 years; (c) Eastern Cooperative Oncology Group performance status less than or equal to 1; (d) Child-Pugh score of A or B; (e) serum creatinine less than or equal to 1.5 mg/dL; and (f) patients undergoing at least 2 weeks of sorafenib treatment. The exclusion criteria were: (a) HCC patients with extrahepatic metastasis; (b) previous history of systemic chemotherapy; (c) LT prior to radioembolization; (d) and benign PVT confirmed by experienced radiologists.

**Fig 1:**
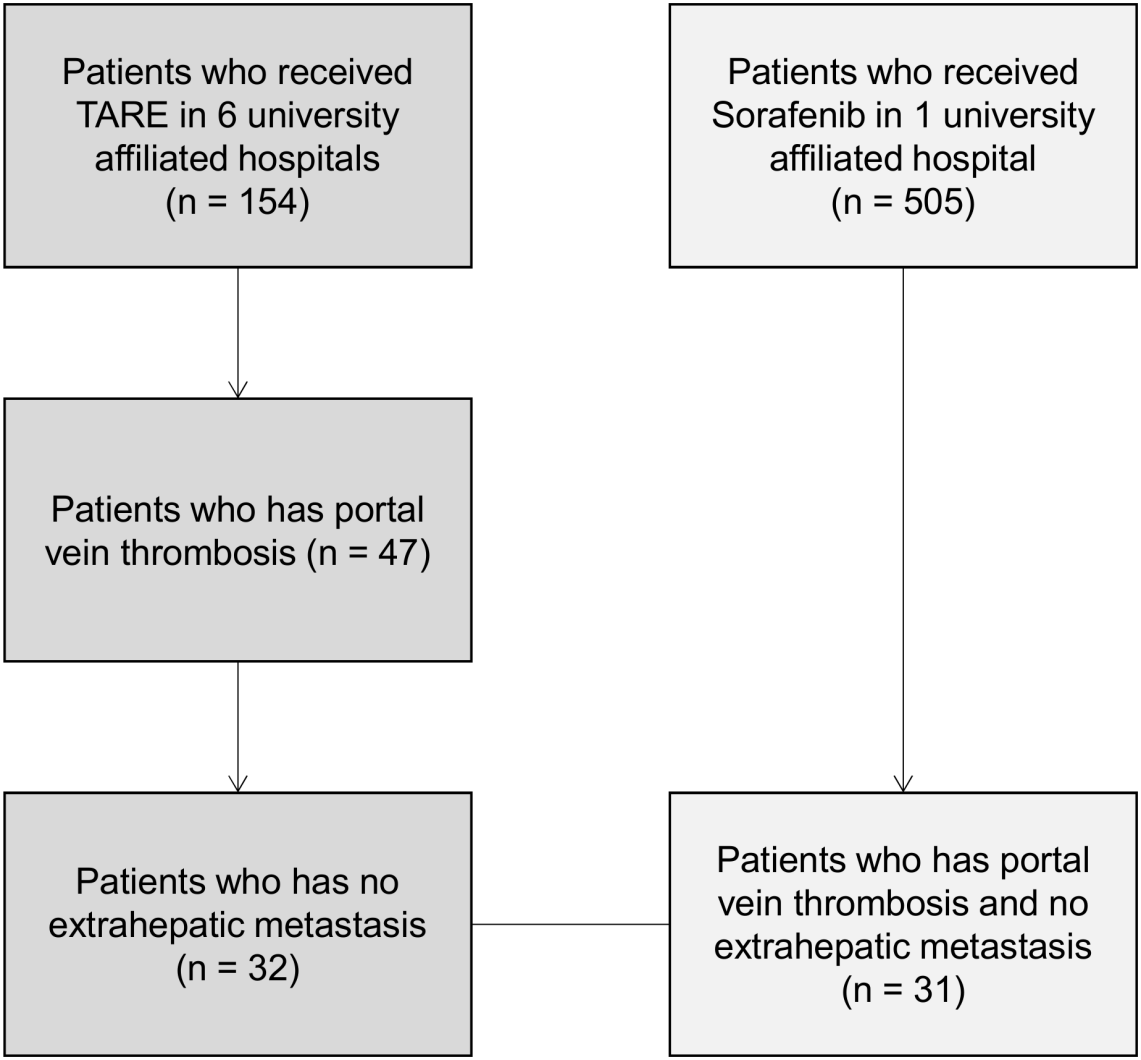
Patient selection.

A total of 154 patients were treated with radioembolization, and, of these, 47 patients had PVT and met the inclusion criteria. Among these, 32 patients did not have extrahepatic metastasis and were selected for the radioembolization group. A total of 505 sorafenib-treated patients were reviewed and 31 patients met the inclusion criteria and were selected for the control group (Fig 1).

### Pre-radioembolization evaluation

Subjects underwent a pre-radioembolization evaluation to evaluate the suitability to undergo radioembolization. Firstly, diagnostic angiography was performed, the aim of the angiography was to assess vascular structures and to evaluate whether there is shunt from liver to other organs. Simultaneously, technetium albumin aggregated scan (MAA) was performed, the aim of the MAA scan was to assess the percentage of lung shunt and to predict the probability of lung complication caused by radioembolization treatment.

### Radioembolization with Y90

A repeat assessment of treatment eligibility was performed within 4 weeks before radioembolization. The volumes of tumor and normal liver were measured. The radiation dose to the tumor was established on the basis of the manufactures protocol, but the treatment dose was determined by each treatment team. Radiation dose outside of the tumor was calculated, and the radiation were not allowed above the maximum dose proposed by the protocol. The decision of treatment, and whether to treat sequentially or by one-step was made by each treatment team. At the treatment period, only resin-based SIR-Spheres microspheres were available in Korea (SirTex Medical Limited, Lane Cove, Australia).

### Sorafenib treatment

Patients in the sorafenib group received a starting dose of sorafenib 400 mg twice daily. In cases of severe adverse events, sorafenib doses were adjusted by each treating physician. Treatments were continued until tumor progression or intolerable toxicity.

### Clinical follow-up and response

In the radioembolization group, dynamic computed tomography was obtained for each patient at 1 and 3 months after radioembolization and then every 3 months. In the sorafenib group, tumor response was assessed in each patient, first after 1 or 2 months, according to the decision of each physician, and then every 3 months. Tumor response and disease progression were evaluated using the modified RECIST criteria. The response evaluation was based on arterial enhancement. HCC showing no arterial enhance was defined as CR; cases with the sum of target lesions reduced by at least 30% were defined as PR. When there were new lesions, or the sum of target lesions increased by at least 20% the response were defined as progressive disease (PD), and all others were defined as stable disease (SD). The sum of CR and PR was defined as the response rate, and the sum of response rate and SD was defined as the disease control rate. Adverse effects were graded according to Common Terminology Criteria for Adverse Events, version 4.03.

### Statistical analysis

Student’s t-test was used to compare pairs of independent continuous variables. The chi-square test or Fisher’s exact test were used to compare variables. Overall survival (OS) and time to progression (TTP) were calculated from the treatment date and death or the final observation date in case of censoring. Univariate analysis was performed using the Cox regressing, and afterwards multivariate Cox regression was performed to estimate the relation of prognostic factors and survival.

To minimize selection bias, we used inverse probability weighting (IPW). Propensity scores were calculated using generalized boosted regression to predict the probability of each patient receiving radioembolization or sorafenib, using 10 pretreatment variables, including age, sex, Child-Pugh score, MELD, existence of ascites, previous treatment, degree of PVT, alpha-fetoprotein, platelet, and ALT. After propensity scores were calculated, radioembolization and sorafenib groups were balanced by means of IPW [28].

All statistical analysis were performed using SPSS software (SPSS version 20.0; SPSS, Chicago, Ill) and R Statistical Software 3.1 (R Foundation for Statistical Computing, Vienna, Austria). A two-sided *P* value <0.05 was considered statistically significant.

## Results

Baseline characteristics of both patients are shown in Table 1. There were no significant differences in age, gender, Child-Pugh class, or prior treatment of HCC. There were significantly more patients with main PVT in the sorafenib group than in the radioembolization group, and the Model for End-stage Liver Disease score was worse in the sorafenib group. After we applied IPW, the baseline characteristics were balanced and both groups did not differ significantly (shown in S1 Table).

**Table 1:** Baseline characteristics of patients with portal vein thrombosis and without extrahepatic metastasis.

	Radioembolization (n = 32)	Sorafenib (n = 31)	P Value
Age (years), Mean ± SD	63.7 ± 11.1	60.3 ± 10.4	0.22
Gender			0.10
Male, *n* (%)	26 (81.3)	30 (96.8)	
Female, *n* (%)	6 (18.7)	1 (3.2)	
Etiology			
HBV, *n* (%) *	23 (71.9)	30 (96.8)	0.01
HCV, *n* (%)	5 (15.6)	0	0.05
Alcoholism, *n* (%)	2 (6.3)	1 (3.2)	1.00
Cryptogenic, n (%)	3 (9.4)	0	0.24
Child-Pugh score, n (%)			0.13
A	28 (87.5)	22 (71.0)	
B	4 (12.5)	9 (29.0)	
Ascites, *n* (%)	5 (15.6)	6 (19.4)	0.75
Prior treatment of HCC, n (%)			0.13
Treatment	10 (31.3)	4 (12.9)	
None	22 (68.7)	27 (87.1)	
Portal vein invasion, *n* (%) **			<0.01
Second-order	12 (37.5)	6 (19.4)	
First-order	20 (62.5)	10 (32.3)	
Main	0	15 (48.3)	
Hepatic vein invasion, n (%)	4 (12.5)	4 (12.9)	0.63
Tumor morphology, n (%)			0.67
Uninodular and extension ≤ 50%	9 (29.0)	6 (19.4)	
Multinodular and extension ≤ 50%	15 (48.4)	17 (54.8)	
Massive or extension > 50%	7 (22.6)	8 (25.8)	
AFP (ng/mL), n (%)			0.82
≤ 20 ng/mL	5 (15.6)	6 (19.4)	
20–200 ng/mL	7 (21.9)	5 (16.1)	
>200 ng/mL	20 (62.5)	20 (64.5)	
Platelet (10^3^/mL), Mean ± SD	168.4 ± 87.9	156.2 ± 81.2	0.59
ALT (IU/L), Mean ± SD **	49.1 ± 49.2	94.7 ± 132.9	<0.01
MELD, Mean ± SD **	5.10 ± 3.62	7.50 ± 2.86	<0.01

SD, stand deviation; MELD, The Model for End-Stage Liver Disease; AFP, Alpha-fetoprotein

* P values < 0.05,

** P values < 0.01

### Treatment responses

The characteristics of radioembolization treatments are summarized in Table 2. Responses to radioembolization and sorafenib treatments are summarized in Table 3. In the radioembolization group, 2 (6.3%), 7 (21.9%), and 7 (21.9%) patients showed CR, PR, and SD, respectively, at 3 months. The response rate was 28.2% (9/32) and the disease control rate was 50.1% (16/32). At 6 months, the response rate was 6.3% (2/32) and the disease control rate was 34.4% (11/32). In the sorafenib group, 0, 1 (3.2%), and 12 (38.7%) patients showed CR, PR, and SD, respectively, at 3 months. The response rate was 3.2% (1/31) and the disease control rate was 41.9% (13/31). At 6 months, the response rate was 3.2% (1/31) and the disease control rate was 19.4% (6/32). Compared to the sorafenib group, the radioembolization group showed a superior response rate at 3 months (P = 0.01).

**Table 2:** Radioembolization treatment characteristics.

	All patients (n = 32)
Target Treatment, n (%)	
Whole liver	4 (12.5)
Right lobe	10 (31.3)
Left lobe	4 (12.5)
Segmental	11 (34.4)
Missing	3 (9.3)
Target tumor volume (ml), mean (range)	511.9 (5.0–1754.9)
Target liver volume (ml), mean (range)	1046.6 (280.0–1845.0)
Whole liver volume (ml), mean (range)	1701.9 (998.3–2522.6)
Total injected activity (GBq), mean (range)	2.08 (0.28–4.93)
Lung shunt (%), mean (range)	9.76 (1.63–35.4)

**Table 3:** Response to treatment.

	3 months			6 months		
	Radioembolization (n = 32)	Sorafenib (n = 31)	P Value	Radioembolization (n = 32)	Sorafenib (n = 31)	P Value
CR, n (%)	2 (6.3)	0	0.49	2 (6.3)	0	0.54
PR, n (%)	7 (21.9)	1 (3.2)	0.05	0	1 (3.2)	0.33
SD, n (%)	7 (21.9)	12 (38.7)	0.10	9 (28.1)	5 (16.1)	1.00
PD, n (%)	12 (37.5)	13 (41.9)	0.59	9 (28.1)	4 (12.9)	1.00
Overall, n (%)	28 (87.5)	26 (83.9)		20 (62.5)	10 (32.3)	
Response rate, n (%)	9 (28.1)	1 (3.2)	0.01*	2 (6.3)	1 (3.2)	1.00
Disease control rate, n (%)	16 (50.0)	13 (41.9)	0.78	11 (34.4)	6 (19.4)	1.00

CR, complete remission; PR, partial remission; SD, stable disease; PD, progressive disease

* P values < 0.05

### Overall survival and time to progression

**Fig 2:**
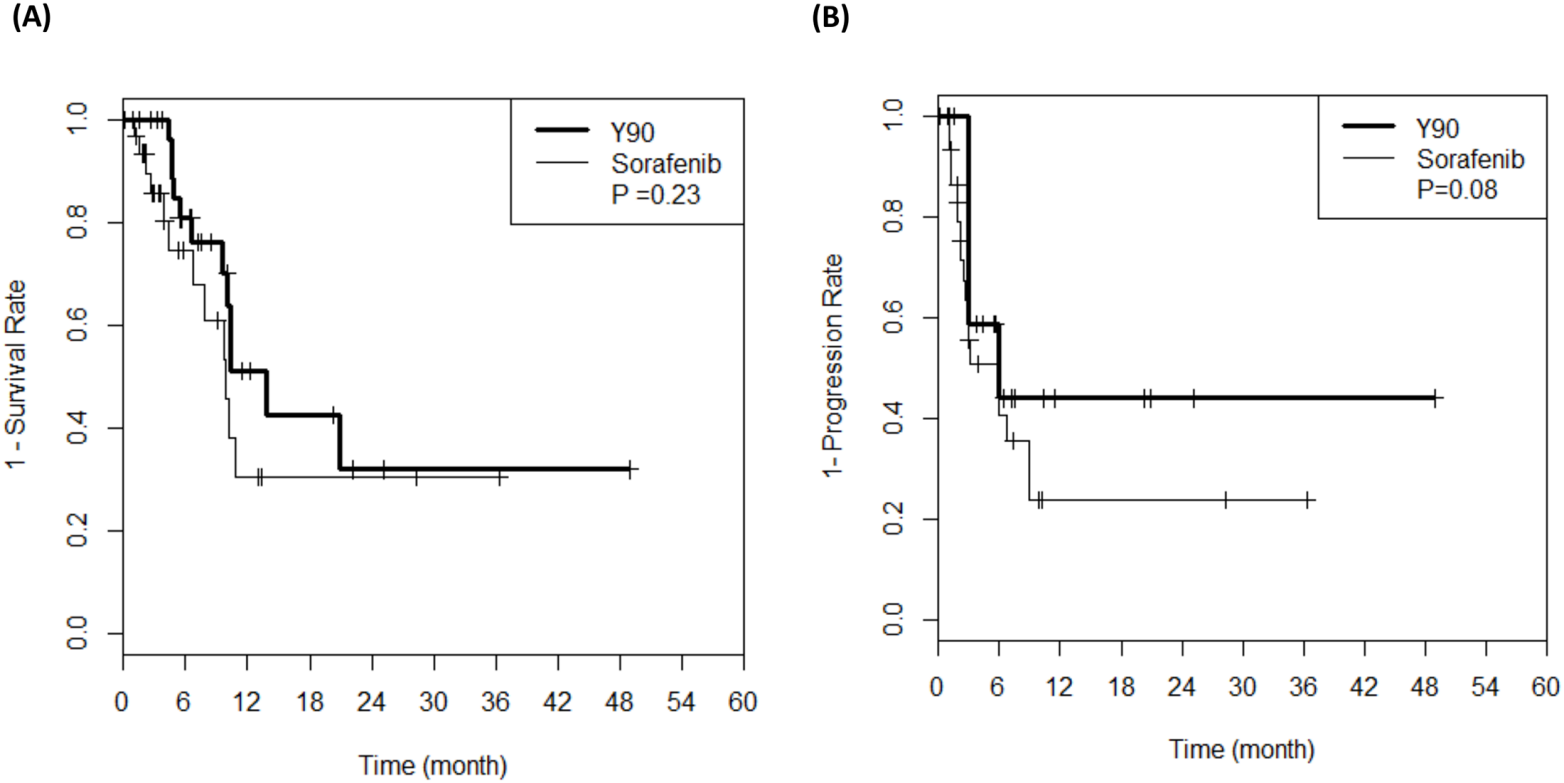
Survival analysis. (A) Overall survival and (B) Time to progression of patients treated with sorafenib and radioembolization.

**Fig 3:**
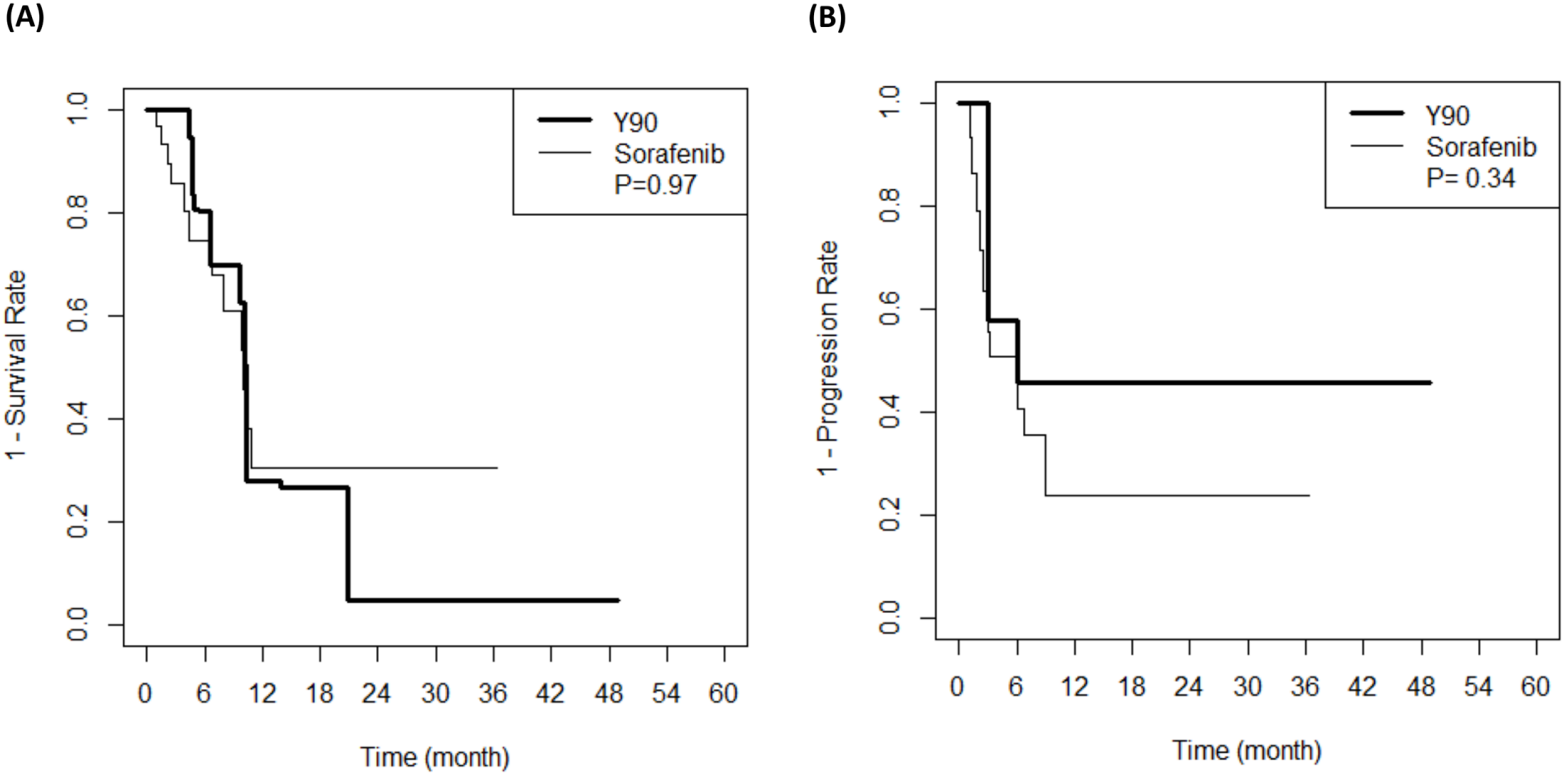
Survival analysis of balanced population after inverse probability weighting. (A) Overall survival and (B) Time to progression of patients treated with sorafenib and radioembolization.

During follow-up, 12 patients died in the radioembolization group and 12 patients died in the sorafenib group. The median OS and TTP of the radioembolization group and the sorafenib group were not significantly different (13.8 months and 10.0 months, P = 0.23; and 6.0 months and 6.0 months, P = 0.08), respectively (Fig 2). No differences in OS (P = 0.97) or TTP (P = 0.34) were observed (Fig 3), even after we applied IPW. The baseline characteristics of the balanced population are shown in the supplemental material (S1 Table).

In the univariate analysis, the OS was significantly associated with the Child-Pugh score and tumor morphology (Table 4). We used these variables in a multivariate Cox analysis, which revealed that a high Child-Pugh score (hazard ratio [HR], 2.75; 95% confidence interval [CI], 1.04–7.29; P = 0.04) and massive tumor morphology (HR, 5.01; 95% CI, 1.00–25.1; P = 0.05) were associated with worse survival.

**Table 4:** Univariate and multivariate cox-regression of patients included.

	Overall Survival—univariate		Overall Survival—multivariate	
Parameter	HR (95% CI)	P Value	HR (95% CI)	P Value
Age	0.97 (0.93–1.02)	0.17		
Sex				
Female	1.24 (0.37–4.23)	0.73		
Treatment				
Sorafenib treatment	1.63 (0.73–3.66)	0.23		
Child-Pugh score				
A	1		1	
B	2.41 (1.03–5.63)	0.04 *	2.75 (1.04–7.29)	0.04 *
Ascites present	1.78 (0.66–4.87)	0.25		
Prior treatment of HCC				
Treatment	1.13 (0.50–2.53)	0.77		
Portal vein invasion				
Second-order	1			
First-order	0.76 (0.32–1.85)	0.55		
Main	0.80 (0.25–2.58)	0.71		
Tumor morphology, n (%)				
Uninodular and extension ≤ 50%	1		1	
Multinodular and extension ≤ 50%	3.57 (0.80–15.8)	0.09	4.23 (0.94–19.6)	0.06
Massive or extension > 50%	5.70 (1.16–28.0)	0.03 *	5.01 (1.00–25.1)	0.05 *
MELD	1.08 (0.97–1.20)	0.16		

HCC, hepatocellular carcinoma; MELD, The Model for End-Stage Liver Disease

* P values < 0.05,

** P values < 0.01

### Toxicity

Among the radioembolization group, the most common adverse effects were nausea or vomiting (21.9%) and abdominal pain (18.8%). In the sorafenib group, the most common adverse effect was diarrhea (13.0%). There were significantly more severe adverse events (grade 3/4) in the sorafenib group than in the radioembolization group (5/31 and 1/32; P < 0.01, respectively; Table 5).

**Table 5:** Adverse effects according to CTCAE ver 4.03.

	Radioembolization		Sorafenib		
	Grade 1/2	Grade 3/4	Grade 1/2	Grade 3/4	P value
Nausea or vomiting, n (%)	7 (21.9)	0	1 (3.2)	1 (3.2)	0.14
Anorexia, n (%)	0	0	1 (3.2)	0	0.07
Diarrhea, n (%)	0	0	2 (6.5)	2 (6.5)	<0.01
Abdominal pain, n (%)	6 (18.8)	0	1 (3.2)	1 (3.2)	0.03
Fever, n (%)	0	0	0	0	-
Weight loss, n (%)	0	0	0	0	-
Bleeding, n (%)	0	0	0	1 (3.2)	<0.01
Splenic infarction, n (%)	0	1 (3.1)	0	0	<0.01

## Discussion

Radioembolization is emerging as a valuable treatment option for PVT. The results of this study indicate that comparable response rates can be achieved with radioembolization and with sorafenib. The median survivals of the radioembolization group (13.8 months) and the sorafenib group (10.0 months) were not significantly different, and CR cases (6.3%) only occurred in the radioembolization group. We applied IPW to overcome imbalances in baseline characteristics, and, after establishing a balanced population, the two treatment groups still showed no significant differences in survival or progression. In fact, the sorafenib group showed significantly more grade 3/4 adverse events than the radioembolization group. The results of our study support the use of radioembolization to treat HCC patients with PVT.

The median survivals reported in our study are comparable with previous studies. Few studies have reported that radioembolization can be delivered with positive outcomes in HCC patients with PVT, and those studies reported median OS ranging from 5.6 to 13.8 months [17, 23–26]. The extent of PVT and Child-Pugh classification are the main prognostic factors that affect survival in HCC. A previous study showed that patients with branch PVT had a better prognosis than patients with main PVT (10.7 vs 9.7 months, respectively) [29], and another study showed that patients with Child-Pugh A disease had a better prognosis than patients with Child-Pugh B7 disease (13.8 vs 6.5 months, respectively). Sub-analyses of the SHARP study and the Asian-Pacific trial revealed that patients with macrovascular vessel invasion had median OS of 8.1 [6] and 5.6 [7] months, respectively. We observed median OS of 10.0 months in the sorafenib group and 13.8 months in the radioembolization group. These findings can be partly explained by the exclusion of HCC patients with extrahepatic metastasis, which is known to be associated with poor prognosis [30]. A recent study comparing radioembolization and sorafenib for intermediate to locally advanced HCC showed median OS of 14.4 months in the sorafenib group and 13.2 months in the radioembolization group [19], which are comparable to the findings of our study. Another study comparing radioembolization and sorafenib for HCC with PVT showed median OS of 8.8 months in the radioembolization group and 6.7 months in the sorafenib group [26], which showed shorter OS compared to our study, but this study included patients with extrahepatic metastasis. Therefore, the existence of extrahepatic metastasis might influenced the OS negatively in this study. Although western guidelines suggest sorafenib as the treatment of choice for HCC patients with PVT [4, 5], the survival gain of sorafenib compared to placebo is minimal, especially in subgroup analyses of vascular invasion. Further, the SHARP trial and the Asian-Pacific trial reported no CR cases. Because of the need for effective treatment options for HCC with PVT, locoregional treatments are frequently performed in real world practice, especially in Asian countries, which is reflected in the Asia-Pacific guidelines [14]. PVT in HCC is associated with both intrahepatic and extrahepatic spread, and using sorafenib to treat PVT as a systemic disease has recognized rationale, and large randomized controlled studies have proved survival gains from this treatment. However, without effective local control, sorafenib is unlikely to control this life-threatening condition effectively, and local control of PVT should be attempted if it is possible and patients are eligible [31]. Patients with locally advanced HCC with no extrahepatic metastasis and vascular invasion only have favorable outcomes compared to patients with HCC with extrahepatic metastasis [30]. This may suggest that, in this specific patient population, PVT can be treated in a locoregional manner. Treating PVT with a locoregional method is less invasive, allows down-staging that can permit LT, and, in certain cases, shows dramatic outcomes compared to sorafenib [31]. The main problem with the use of TACE to treat patients with PVT is that embolization of PVT can cause acute decompensation. Compared to TACE, radioembolization shows better arterial patency and less embolization. For this reason, radioembolization is believed to have a lower risk of post-embolization syndrome and a better safety profile than sorafenib [22]. Accordingly, in our study, the radioembolization group showed significantly less severe adverse events, and no cases of acute hepatic decompensation occurred in either the radioembolization group or the sorafenib group.

Radioembolization might be an important tool in the treatment of HCC patients with PVT. Two randomized control studies are currently ongoing to clarify this issue: the SARAH study (Sorafenib versus Radioembolization in Advanced Hepatocellular carcinoma, ClinicalTrials.gov identifier NCT01482442) and the STOP HCC study (Efficacy Evaluation of TheraSphere in Patients With Inoperable Liver Cancer, ClinicalTrials.gov identifier NCT01556490). As previously mentioned, a recent Italian study found that radioembolization and sorafenib showed comparable outcomes for intermediate to locally advanced HCC [19], and a previous Spanish study found that radioembolization showed superior outcomes for HCC with PVT [26].

The strength of our study compared to previous studies is that we focused on patients with locally advanced HCC with PVT. Therefore, the results of this study offer direct evidence to support treating HCC patients with PVT with radioembolization. Although a recent study compared radioembolization in HCC patients with PVT, the results of our study provides valuable data in the debatable issue. Since this study was performed in an Asian population, it also provides validation for radioembolization in a setting where hepatitis B is the predominant cause of liver cirrhosis.

There are several limitations of our study. First, the study is performed with retrospective design. In order to overcome this weakness, we performed IPW and matched the characteristics of the sorafenib and the radioembolization groups. Second, the study population was relatively small to reach definitive conclusions and additional stratification based on important clinical variables such as extent of PVT could not be performed. Ongoing or future clinical trials with a sufficient number of patients would allow definitive conclusions to be drawn. Third, although we excluded benign PVTs confirmed by experienced radiologists, some PVTs we treated as tumor invasion may be potentially benign PVTs. Fourth, most radioembolization treated patients followed up at fixed intervals, but sorafenib patients followed up at intervals depending to each physician’s decision. This may make bias at TTP analysis, but the primary endpoint was overall survival, and this bias is not critical in this study.

Radioembolization showed comparable response rates to sorafenib in HCC patients with PVT. We suggest that radioembolization should be considered a new treatment option in this population. Results of ongoing, randomized, controlled studies on this subject will yield further evidence of our findings. To date, radioembolization has been an important option for HCC patients with PVT, and further studies are needed to define which subgroups will benefit from radioembolization or sorafenib.

## Supporting Information

S1 Table Baseline characteristics of balanced population. (DOCX)
