# Supplementary material for: Radioembolization Is a Safe and Effective Treatment for Hepatocellular Carcinoma with Portal Vein Thrombosis: A Propensity Score Analysis
Source: PLoS One. 2016 May 5;11(5):e0154986. doi: 10.1371/journal.pone.0154986 (PMC4858257; doi:10.1371/journal.pone.0154986)
Supplement: S1 Table — (DOCX) [file pone.0154986.s001.docx]

**S1 Table.** **Baseline characteristics of balanced population.**

|  | Radioembolization (n =32) (weighted) | Sorafenib (n = 31) (weighted) | Standard effect size (weighted) | p-value (weighted) |
| --- | --- | --- | --- | --- |
| Age, % |  |  |  | 0.96 |
| ≤ 60 | 48.4 | 49.2 |  |  |
| > 60 | 51.6 | 50.8 |  |  |
| Gender, % |  |  |  | 0.13 |
| Male | 96.8 | 85.3 |  |  |
| Female | 3.2 | 14.7 |  |  |
| Child-Pugh score , % |  |  |  | 0.22 |
| A | 71.0 | 86.9 |  |  |
| B | 29.0 | 13.1 |  |  |
| Ascites, % | 19.4 | 8.2 |  | 0.16 |
| MELD, Mean ± SD | 7.50 ± 2.86 | 6.88 ± 4.06 | 0.08 | 0.88 |
| Previous treatment, % | 61.3 | 35.1 |  | 0.26 |
| Portal vein invasion, *%* |  |  |  | 0.09 |
| Second-order | 19.4 | 34.3 |  |  |
| First-order | 32.3 | 65.7 |  |  |
| Main | 48.4 | 0 |  |  |
| AFP (ng/mL), % |  |  |  | 0.79 |
| ≤ 20 ng/mL | 19.4 | 15.4 |  |  |
| 20-200 ng/mL | 16.1 | 10.5 |  |  |
| >200 ng/mL | 64.5 | 74.1 |  |  |
| Platelet (ng/mL), Mean ± SD | 156.2 ± 81.2 | 169.8 ± 80.1 | -0.17 | 0.61 |
| ALT (IU/L), Mean ± SD | 94.8 ± 132  .9 | 75.6 ± 63.6 | 0.14 | 0.53 |

MELD, The Model for End-Stage Liver Disease; SD, standard deviation;
